# Association of CSF Proteome with CSF P‐tau181 levels and other biomarkers of Alzheimer's Disease

**DOI:** 10.1002/alz.091398

**Published:** 2025-01-09

**Authors:** Min N Qiao, Alex N Rookyard, Lipi N Das, Marielba Zerlin‐Esteves, Dolly Reyes‐Dumeyer, Annie J. Lee, Rafael A. Lantigua, Martin Medrano, Diones Rivera Mejia, Lawrence S. Honig, Lewis M Brown, Richard Mayeux, Badri N. Vardarajan

**Affiliations:** ^1^ Columbia University, New York, NY USA; ^2^ Columbia University, Dobbs Ferry, NY USA; ^3^ Columbia University Irving Medical Center, New York, NY USA; ^4^ G. H. Sergievsky Center, Vagelos College of Physicians and Surgeons, Columbia University, New York, NY USA; ^5^ The Taub Institute for Research on Alzheimer’s Disease and the Aging Brain, Vagelos College of Physicians & Surgeons, Columbia University, New York, NY USA; ^6^ Department of Neurology, Columbia University Medical Center, New York, NY USA; ^7^ School of Medicine, Pontificia Universidad Catolica Madre y Maestra, Santiago Dominican Republic; ^8^ CEDIMAT, Santo Domingo, Santo Domingo Dominican Republic; ^9^ School of Medicine, Universidad Nacional Pedro Henriquez Ureña, Santo Domingo Dominican Republic; ^10^ Taub Institute for Research on Alzheimer’s Disease and the Aging Brain, Vagelos College of Physicians and Surgeons, Columbia University, New York, NY USA; ^11^ Department of Neurology, Vagelos College of Physicians and Surgeons, Columbia University, and the New York Presbyterian Hospital, New York, NY USA; ^12^ The Gertrude H. Sergievsky Center, College of Physicians and Surgeons, Columbia University, New York, NY USA; ^13^ Departments of Neurology, Psychiatry, and Epidemiology, Gertrude H. Sergievsky Center, The Taub Institute for Research on Alzheimer’s Disease and the Aging Brain, Vagelos College of Physicians and Surgeons, Columbia University, New York, NY USA; ^14^ Gertrude H. Sergievsky Center, Vagelos College of Physicians & Surgeons, Columbia University, New York, NY USA; ^15^ Taub Institute for Research on Alzheimer’s Disease and the Aging Brain, Columbia University, New York, NY USA; ^16^ Department of Neurology, New York, NY USA

## Abstract

**Background:**

We investigated the relationship between the cerebrospinal fluid (CSF) proteome in Alzheimer’s disease (AD) and the clinical and biomarker‐assisted diagnoses.

**Methods:**

CSF was collected in 500 individuals of non‐Hispanic white, African Americans, and Caribbean Hispanic individuals from Dominican Republic and New York City. CSF biomarkers of AD were measured including P‐tau181, Aβ40, Aβ42, total‐tau, neurofilament light chain (NfL) and glial fibrillary acidic protein (GFAP). CSF was depleted of abundant proteins followed by precipitation, cysteine reduction/alkylation, and proteolytic cleavage by trypsin. Peptides were measured using a Q Exactive HF mass spectrometer (Thermo Scientific). Association of individual and co‐abundant modules of proteins were tested with the clinical diagnosis of AD, as well as biologically defined AD pathological process based on CSF P‐tau181 and other biomarker levels.

**Results:**

We detected 1030 protein groups (with at least one peptide per protein), yielding an overall data completeness value of 97%. CSF levels of 41 proteins were significantly associated with P‐tau181 levels after multiple testing correction. Notably, phospholipase D3 (PLD3, p=2.41E‐09), APOE (p=4.25e‐08) and osteopontin (OSTP, p=1.4E‐16) were increased and ceruloplasmin (CERU, p=2.72E‐07) decreased among individuals with high P‐tau181 levels. These proteins were also associated with CSF Aβ42/Aβ40 ratio and total Tau levels but not with NfL. OSTP was also associated with CSF levels of GFAP (p=1.32e‐05). We did not identify any protein association with clinical AD. Among proteins associated with P‐tau181 levels, pathways related to axon development (p=2.4E‐12), axonogenesis (p=1.45E‐11) and regulation of axonogenesis (p=5.1E‐09) were enriched.

**Conclusion:**

Unbiased profiling of circulating CSF proteins identified key proteins associated with β‐amyloid and phosphorylated tau pathology. Biologically based diagnostic criteria may aid in the identification of unique pathogenic mechanisms.

